# The mechanism of action of the combination of *Astragalus membranaceus* and *Ligusticum chuanxiong* in the treatment of ischemic stroke based on network pharmacology and molecular docking

**DOI:** 10.1097/MD.0000000000029593

**Published:** 2022-07-15

**Authors:** Tianyue Wang, Xinyu Jiang, Yanmin Ruan, Lin Li, Lisheng Chu

**Affiliations:** a The 2nd Clinical Medical College, Zhejiang Chinese Medical University, Hangzhou, China; b The 1st Clinical Medical College, Zhejiang Chinese Medical University, Hangzhou, China; c Department of Physiology, Zhejiang Chinese Medical University, Hangzhou, China.

**Keywords:** *Astragalus membranaceus*, ischemic stroke, *Ligusticum chuanxiong*, molecular docking, network pharmacology

## Abstract

Since 1990, the incidence of stroke has been rising to become the second leading cause of death in the world, posing a huge burden and challenge to society and families.

*Astragalus membranaceus* and *Ligusticum chuanxiong* (A&L) have been used as traditional Chinese medicine (TCM) prescriptions to treat and prevent the occurrence of ischemic stroke (IS), but their mechanism of action on the disease has not been fully elucidated. The main objective of this study was to reveal the pharmacological mechanism of A&L in the treatment of IS and to perform preliminary validation.

The active ingredients of A&L were obtained from the systematic pharmacology platform of traditional Chinese medicine (TCMSP) database, whereas the genes of IS were obtained from 2 major databases, DrugBank and GeneCards. Cytoscape_v3.8.2 was used to construct the TCM-active ingredient and TCM-active ingredient-cross-target-disease relationship maps, and the MCODE plug-in was used to obtain the core genes, whereas the protein-protein interaction maps were obtained from the STRING database. The results of gene ontology and Kyoto encyclopedia of genes and genomes enrichment were obtained using the Hiplot online tool, and the small molecules in the relevant signalling pathways were verified by molecular docking using AutoDock.

A&L contained a total of 26 eligible active ingredients, sharing 161 common targets with IS. A total of 58 core genes with 1326 edges were obtained using the MCODE plug-in. Gene ontology and Kyoto encyclopedia of genes and genomes enrichment results showed association with interleukin-17 signaling pathway, lipid and atherosclerosis, tumor necrosis factor signaling pathway, and Toll-like receptor signaling pathway, which mainly mediates the development of inflammatory responses. Furthermore, molecular docking was conducted and most of the components were found to have good binding to the receptors.

This study demonstrates that A&L can be used to treat IS by controlling the inflammatory response through multiple targets and multiple pathways, and provides a reference for subsequent trials.

## 1. Introduction

According to the latest findings of the World Stroke Organisation, the stroke will remain the world’s second leading cause of death in the coming years, with global stroke costing >$721 billion. Stroke cases have also been on the rise each year since 1990, placing a huge economic burden on society and healthcare.^[1]^ Stroke is a cerebrovascular disease usually caused by the rupture or blockage of a blood vessel in the brain. Clinically, stroke is usually characterized by hemorrhage or infarction in the corresponding brain area, resulting in partial neurological impairment.^[2]^ Ischemic stroke (IS) accounts for the largest proportion of all stroke types, about 80% or more.^[3]^ After the development of an IS, blood may be irreversibly damaged in some brain regions due to the inability to properly supply the brain with sufficient oxygen and glucose. The disruption of energy metabolism causes inactivation of the associated ionic pumps and channels as well as an increase in the concentration of toxic excitatory neurotransmitters, and this series of physiological changes can lead to neuronal death.^[4]^ In addition, the inflammatory response that occurs after the IS onset also continues to exacerbate the neurological impairment in the brain, such as the activation of microglia that increases the permeability of the blood–brain barrier and the infiltration of peripheral immune cells.^[5]^

At present, recombinant tissue plasminogen activator remains the only clinically approved drug by the United States Food and Drug Administration, but its use is limited to a small population, and reperfusion therapy is still associated with certain risks.^[6]^

Traditional Chinese medicine (TCM) involves multiple herbal medicines combined with each other to treat diseases. Compared with single-drug treatment modalities, TCM can improve treatment outcomes. In China, TCM believes that stroke is caused by Qi deficiency and blood stasis, so benefiting Qi and removing stasis is the main treatment for this disease.^[7]^ Buyang Huanwu Decoction, Dan Hong Injection and Qing Kai Ling Injection are all excellent formulas for the treatment of cerebral ischemia in Chinese medicine, which have the effects of activating blood circulation, promoting blood circulation, and clearing heat and detoxification, and can promote the therapy of stroke recovery, while also having certain anti-inflammatory effects to alleviate the damage caused by reperfusion.^[8–10]^

*Astragalus membranaceus* and *Ligusticum chuanxiong* (A&L) are widely used in TCM prescriptions for the treatment of cardiovascular and cerebrovascular diseases. *Astragalus membranaceus* (AM) contains many active molecules such as Astragaloside, which has been found to promote neural stem cell proliferation by affecting the Akt signaling pathway and improving spatial memory,^[11]^ as well as interfering with AMP-activated protein kinase and nuclear factor-κB signaling pathways to reduce inflammation and excessive immune response.^[12]^ *Ligusticum chuanxiong* (LC) has been used in TCM to prevent the occurrence of stroke, and relevant clinical trial data have confirmed the scientific validity and reliability of the drug.^[13]^ The phthalates and alkaloids in LC extract play a major role in its pharmacological activities, including anticerebral ischemia, antimyocardial ischemia, vascular protection, antithrombosis, antihypertension, antiatherosclerosis, antispasmodic, anti-inflammatory, anticancer, antioxidant, and antiasthma.^[14]^

**Figure 1.**
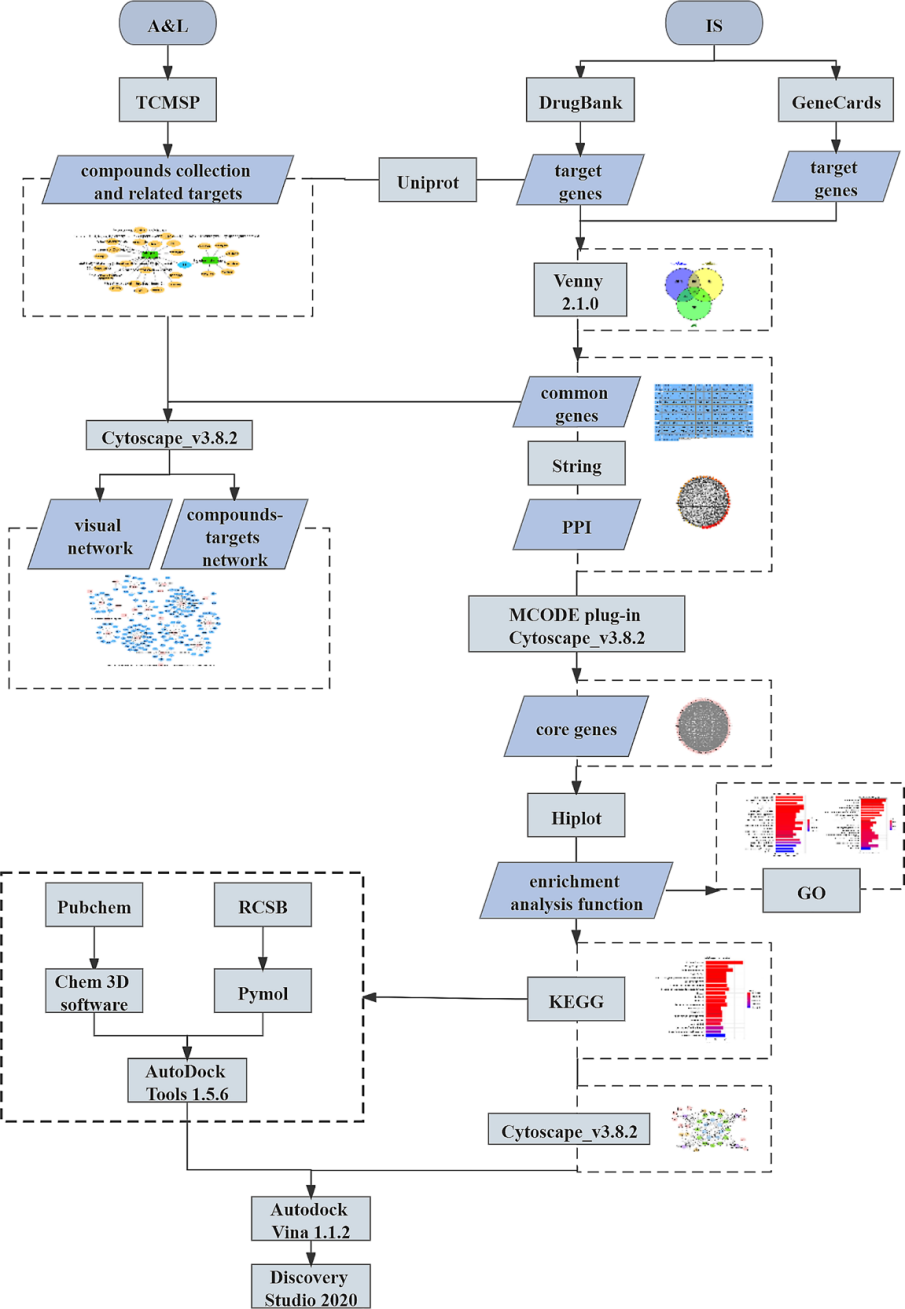
Network pharmacology analysis workflow. A&L = *Astragalus membranaceus* and *Ligusticum chuanxiong*; GO = gene ontology; IS = ischemic stroke; KEGG = Kyoto encyclopedia of genes and genomes; PPI = Protein-Protein Interaction; TCMSP = systematic pharmacology platform of traditional Chinese medicine.

The combination of A&L for the treatment of IS is a traditional approach in Chinese medicine to deal with such diseases. Some experiments have shown that AM extract combined with Tetramethylpyrazine can effectively treat the inflammatory response brought about by cerebral ischemia in rats.^[15]^ However, the mechanism of therapeutic action associated with the combination of the 2 has not yet been elucidated. In recent years, the application of network pharmacology in the study of Chinese herbal prescriptions has become more and more widespread, through the construction and analysis of networks to reveal the targets corresponding to the drug treatment diseases, and finally validated by molecular docking.^[16]^ This approach was also used in this paper, aiming to reveal the mechanism of action of the combination of A&L in the treatment of IS and to validate it by molecular docking. The detailed workflow diagram of the study is presented in Figure 1.

## 2. Materials and Methods

### 2.1. Establishment of a database of Chinese herbal medicine AM, LC, and diseases of ischemic stroke

A total of 3 existing databases, systematic pharmacology platform of traditional Chinese medicine (TCMSP) (https://old.tcmsp-e.com/tcmsp.php), DrugBank (https://go.drugbank.com/), and GeneCards (https://www.genecards.org/) are required for the creation of the Chinese medicine and disease databases.

The components of A&L were searched in TCMSP, and based on 2 ADME-related models, including oral bioavailability (OB) and drug-likeness (DL), the effective active compounds of both were screened, and the potential targets of action of the active components were collected separately to build a database of Chinese herbal medicines.

Two major databases, GeneCards (https://www.genecards.org/) and DrugBank (https://www.drugbank.ca/), use ischemic stroke as a keyword for collecting disease-related targets.

### 2.2. Chinese herbal-active ingredient relationship diagram

Cytoscape_v3.8.2 is visualization software that clearly represents network relationships by building network graphs. A&L were imported into Cytoscape_v3.8.2 with the database of active ingredients obtained by selection, and a visual network was obtained, in which the nodes represent each individual herbal medicine and active ingredient, and the lines emanating from the nodes are called degrees, representing the relationship between them.

### 2.3. Active ingredient-cross-target-disease relationship diagram of Chinese medicine

First, the targets derived from TCMSP were converted to a common format via Uniprot (https://www.uniprot.org/). Second, the A&L database and IS database were submitted at Venny 2.1.0 online website (https://bioinfogp.cnb.csic.es/tools/venny/index.html) to produce Venn diagrams to derive the cross-targets of TCMSP and diseases. The active ingredients corresponding to the cross-targets were extracted from the A&L database and imported into Cytoscape_v3.8.2 for visualization and analysis.

### 2.4. Protein-protein interaction relationship network

The String database (https://cn.string-db.org/) is a protein interaction relationship retrieval tool that provides interaction relationships based on the confidence score, as well as other ancillary information, such as the availability of protein domains and 3D structures. All protein interaction data are weighted, integrated, and have a calculated reliability value. Import the cross-targets of A&L and IS into the String database and select the Species as “Homo sapiens” to get the protein-protein interaction (PPI) network. Export as “tsv” file.

### 2.5. Analysis of cross-target PPI networks and construction of core networks

The Cytoscape software can be used not only to build visual images but also to perform some level of data analysis using tools such as CytoHubba, Maximal clique centrality (MCC) and MCODE. Import PPI data files exported from the String database into Cytoscape_v3.8.2 to visualize the PPI network. The MCODE plug-in enables clustering in huge protein or gene networks to build functional modules, which can be used to analyze and export core networks from PPI networks of cross-targets. In addition, the top 50 targets in the PPI relationship network are visualized by CytoHubba and MCC plug-in.

### 2.6. Gene ontology and Kyoto encyclopedia of genes and genomes pathway enrichment analysis

The gene ontology (GO) database can be used to analyze target genes at the level of molecular function and biological process (BP) of cellular components, and the Kyoto encyclopedia of genes and genomes (KEGG) database can be used to represent the signaling pathways in which the target genes are located.

The core targets in the core network were imported via the GO/KEGG enrichment analysis function in the Hiplot online tool (https://hiplot.com.cn/), and the KEGG database was selected with the corresponding parameters set, where the *P* threshold was .01. A bubble plot or histogram can be derived for visual analysis and exported as “xlsx” file. In the bar chart, the smaller the *P*-value, the higher the enrichment; the longer the bar, the more genes are enriched.

### 2.7. Molecular docking

After KEGG enrichment, the relevant important signaling pathways were selected and the relevant genes enriched in these signaling pathways were exported and summarized. The database was then used to retrace these genes back to the relevant active small molecules and Cytoscape_v3.8.2 was used for further visualization. The genes corresponding to multiple signaling pathways were screened and then validated by molecular docking.

Molecular docking uses computer technology to predict ligand-target interactions or to describe structure-activity relationships at the molecular level.^[17]^ We downloaded the structures of compounds in SDF format from the Pubchem database (https://pubchem.ncbi.nlm.nih.gov/) and then converted the structures from SDF to MOL2 format using Chem 3D software. Related protein structures were downloaded in PDB format from the RCSB database. Using Pymol software to remove solvent molecules and ligands while using AutoDock Tools 1.5.6 software to add hydrogen, calculate charges, assign atom types, etc. Finally, molecular docking was conducted using Autodock Vina 1.1.2, and the results were visualized by using Discovery Studio 2020 to present the 10 combinations with the lowest binding energy as pictures.

## 3. Results

### 3.1. Active compounds and targets

**Figure 2.**
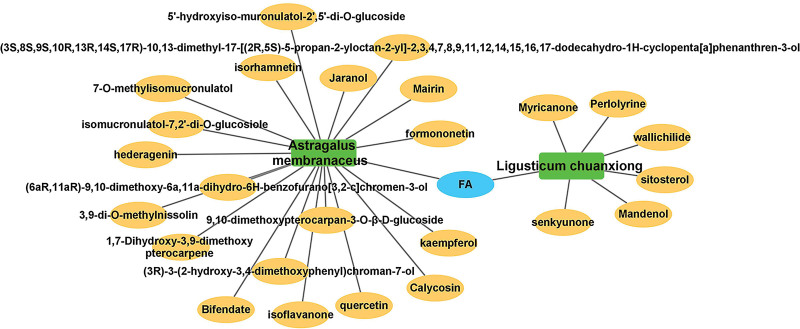
A&L herbal-active ingredient network: green represents herb; orange nodes represent active ingredients unique to both; blue nodes represent active ingredients common to both. A&L = *Astragalus membranaceus* and *Ligusticum chuanxiong.*

Through TCMSP, with OB ≥ 30% and DL ≥ 0.18 as the screening conditions, a total of 26 active compounds were screened, 20 for AM and 7 for LC, with FA being the common active compound for both (Table 1); 220 potential action targets, of which AM contains 218 targets, which shows that AM has a better effect than LC. The correspondence between herbal medicines and active ingredients was imported into Cytoscape_v3.8.2 for visual analysis, resulting in a herbal medicine - active ingredient relationship map (Fig. 2). A total of 3848 potential targets of action associated with IS were collected using the 2 existing available databases, DrugBank and Genecards.

**Table 1:** Potentially active compounds in A&L.

No.	Components	OB (%)	DL	Herb
MOL000211	Mairin	55.38	0.78	AM
MOL000239	Jaranol	50.83	0.29	AM
MOL000296	Hederagenin	36.91	0.75	AM
MOL000033	(3S,8S,9S,10R,13R,14S,17R)-10,13-dimethyl-17-[(2R,5S)-5-propan-2-yloctan-2-yl]-2,3,4,7,8,9,11,12,14,15,16,17-dodecahydro-1H-cyclopenta[a]phenanthren-3-ol	36.23	0.78	AM
MOL000354	Isorhamnetin	49.6	0.31	AM
MOL000371	3,9-di-O-methylnissolin	53.74	0.48	AM
MOL000374	5′-hydroxyiso-muronulatol-2′,5′-di-O-glucoside	41.72	0.69	AM
MOL000378	7-O-methylisomucronulatol	74.69	0.3	AM
MOL000379	9,10-dimethoxypterocarpan-3-O-β-D-glucoside	36.74	0.92	AM
MOL000380	(6aR,11aR)-9,10-dimethoxy-6a,11a-dihydro-6H-benzofurano[3,2-c]chromen-3-ol	64.26	0.42	AM
MOL000387	Bifendate	31.1	0.67	AM
MOL000392	Formononetin	69.67	0.21	AM
MOL000398	Isoflavanone	109.99	0.3	AM
MOL000417	Calycosin	47.75	0.24	AM
MOL000422	Kaempferol	41.88	0.24	AM
MOL000438	(3R)-3-(2-hydroxy-3,4-dimethoxyphenyl)chroman-7-ol	67.67	0.26	AM
MOL000439	isomucronulatol-7,2′-di-O-glucosiole	49.28	0.62	AM
MOL000442	1,7-dihydroxy-3,9-dimethoxy pterocarpene	39.05	0.48	AM
MOL000098	Quercetin	46.43	0.28	AM
MOL000433	FA	68.96	0.71	AM, LC
MOL001494	Mandenol	42	0.19	LC
MOL002135	Myricanone	40.6	0.51	LC
MOL002140	Perlolyrine	65.95	0.27	LC
MOL002151	Senkyunone	47.66	0.24	LC
MOL002157	Wallichilide	42.31	0.71	LC
MOL000359	Sitosterol	36.91	0.75	LC

A&L = *Astragalus membranaceus* and *Ligusticum chuanxiong*; AM = *Astragalus membranaceus*; DL = drug-likeness; LC = *Ligusticum chuanxiong*; OB = oral bioavailability.

**Figure 3.**
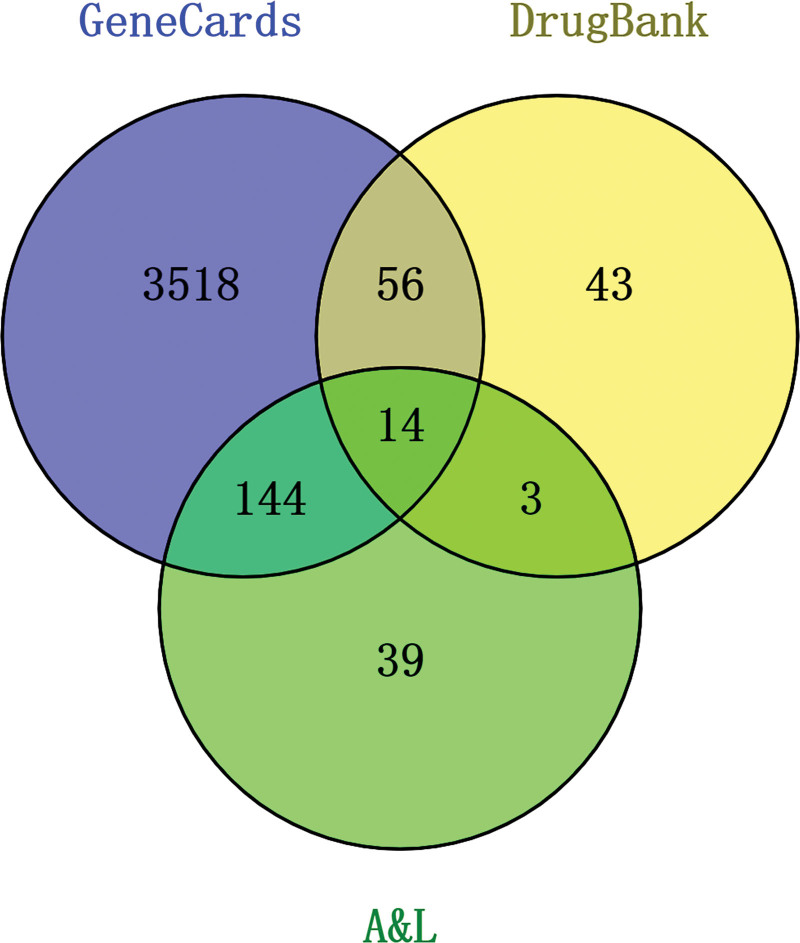
Cross-target Venn diagram of A&L and IS. A&L = *Astragalus membranaceus* and *Ligusticum chuanxiong*; IS = ischemic stroke.

After completing the harmonization of the target format using Uniprot, A&L was used to represent A&M, and the Venny 2.1.0 online tool was used to analyze the target data of Chinese herbs and diseases, construct the Venny diagram, and obtain 161 cross-targets of A&L and IS, as shown in Figure 3.

### 3.2. Common target-active ingredient network for IS and A&L

**Figure 4.**
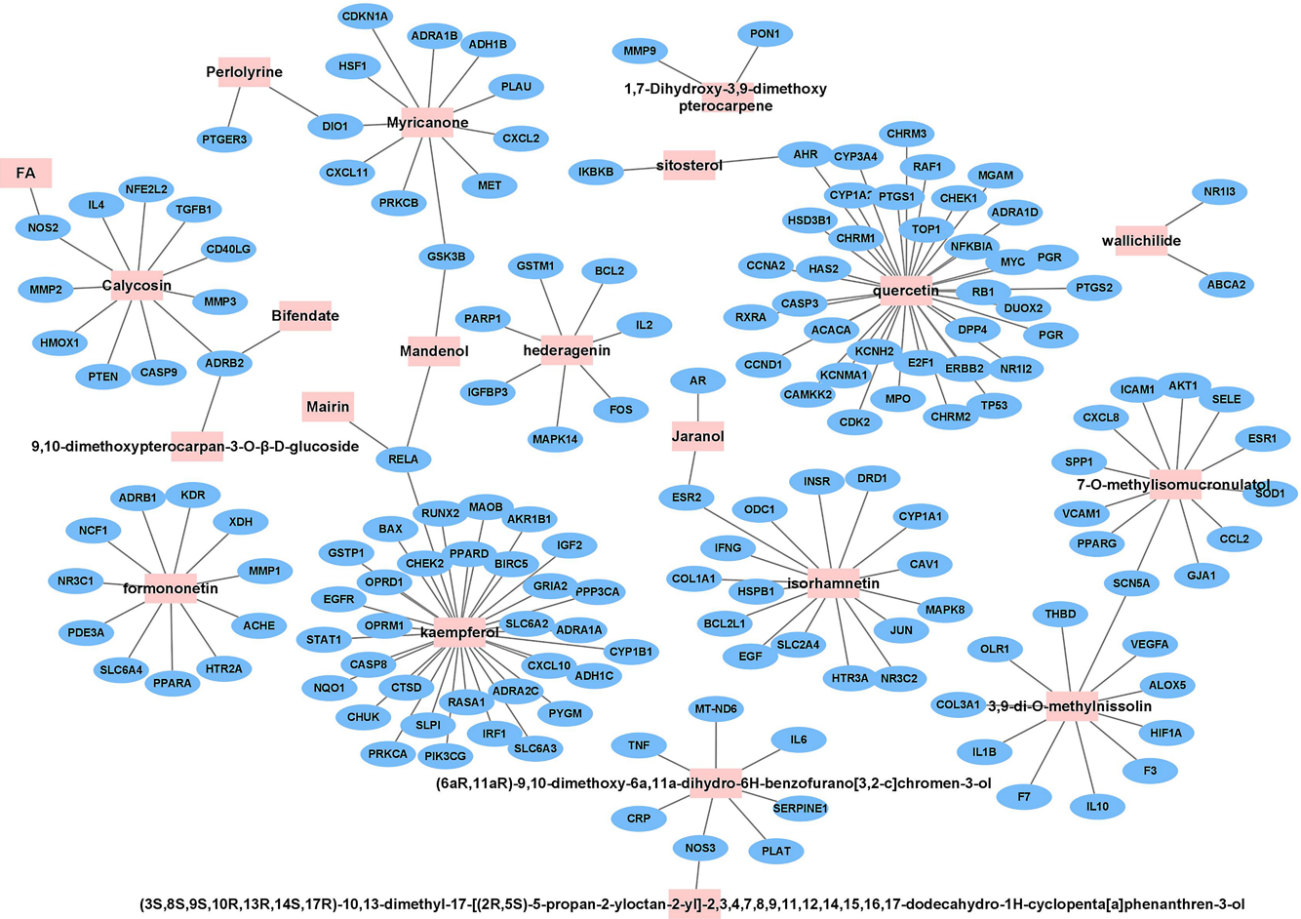
Cross-target-active ingredient network: pink nodes are active ingredients; blue nodes are cross-targets.

The 161 cross-targets were saved in a separate database as an “xlsx” file, and the active ingredients corresponding to these targets were filtered from the established A&L database to establish common target-active ingredient correspondence, which was imported into Cytoscape_v3.8.2 to build a visual relationship network (Fig. 4), facilitating subsequent analysis of the active ingredients in TCM that play a key role in IS using molecular docking.

### 3.3. PPI network and core network

**Figure 5.**
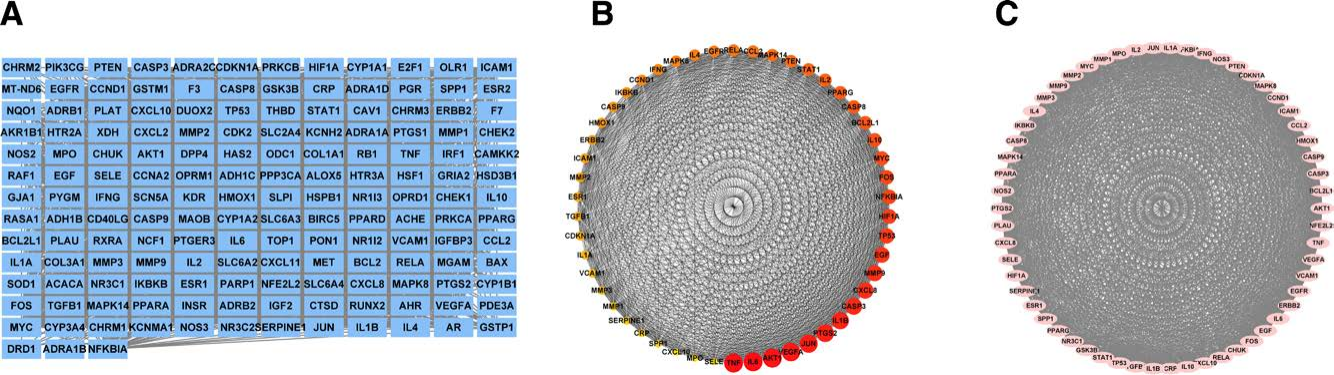
Cross-targeting analysis of A&L and IS. (A) The blue node grid layout is shown for the cross-target PPI network. (B) The cross-target points in the top 50 of the PPI network in terms of degree, the larger the circle, the darker the color, and the higher the degree. (C) The circle diagram surrounded by pink nodes is the core network. A&L = Astragalus membranaceus and Ligusticum chuanxiong; IS = ischemic stroke; PPI = protein-protein interaction.

The 161 cross-targets were imported into String for PPI analysis and the short-expression “tsv” file was selected for further analysis using Cytoscape_v3.8.2. From Figure 5A, it can be seen that only 159 targets were involved in the interaction, with a total of 2964 edges, and ABCA and DIO1 have almost no effect in the protein network.

Analysis of the network using CytoHubba in Cytoscape and the MCC algorithm produced Figure 5B, where larger circles and darker colors represent a more important role in the PPI map, with the top 5 scores being tumor necrosis factor (TNF), interleukin (IL)6, AKT1, VEGFA, and JUN, symbolizing that they may play a key role in diseases such as IS.

Using the MOCDE plug-in for modularity analysis, a core network with a score of 46.526 was selected for this study, containing 58 targets and 1326 edges, as shown in Figure 5C, with the core targets exported as files and saved.

### 3.4. GO and KEGG pathway enrichment analysis

**Figure 6.**
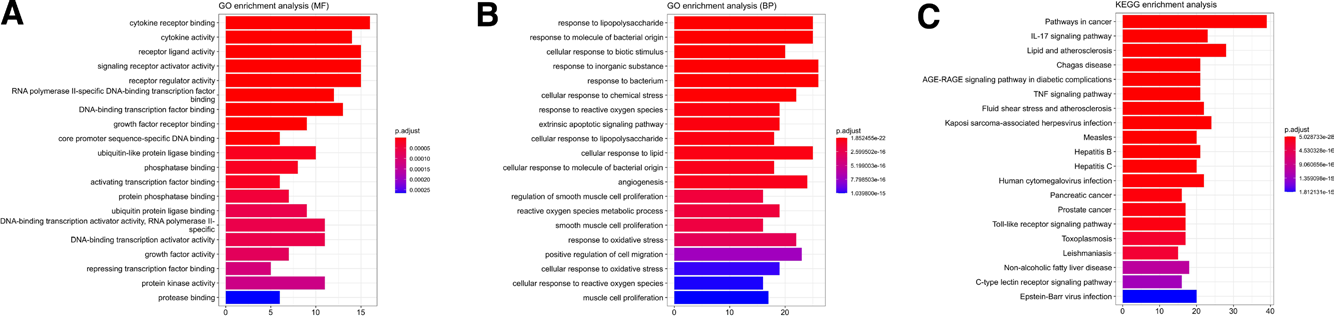
Histogram of the top 20 GO and KEGG pathway enrichment analysis. The GO enrichment results are MF (A) and BP (B), and the KEGG pathway enrichment results are (C). BP = biological process; GO = gene ontology; IL = interleukin; KEGG = Kyoto encyclopedia of genes and genomes; MF = molecular function; TNF = tumor necrosis factor.

The GO enrichment results are shown in Figure 6A and B, and the KEGG signaling pathway enrichment results are shown in Figure 6C. The BPs involved were mainly focused on responses to bacteria-derived molecules, lipopolysaccharides, and biological stimuli.

### 3.5. Molecular docking validation results

**Figure 7.**
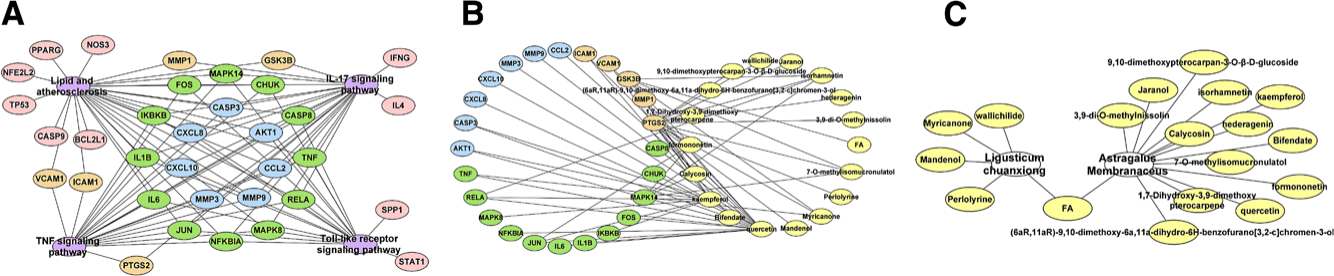
Analysis of the correlation of core signaling pathways, targets and active ingredients. (A) Key signaling pathways and target genes of A&L and IS cross-targets: pink nodes represent target genes contained in one signaling pathway, orange nodes contain in 2 signaling pathways, blue nodes contain in 3 signaling pathways, green nodes contain in 4 signaling pathways; purple nodes represent signaling pathways. (B) Key target genes and active ingredients of crossover genes: the circle on the left represents the crossover target, and the circle on the right represents the active ingredient; the yellow nodes represent the active ingredients of herbal medicines corresponding to the target, and the other color nodes correspond to the above figure. (C) Key components and herbs of crossover genes: yellow nodes represent the active components of herbal medicines, and white nodes represent the herbal medicines corresponding to the targets.

When selecting the signaling pathways to be studied, based on the column length and *P*-value size, as well as the direction of the study, the final selection of IL-17 signaling pathway, lipid and atherosclerosis, TNF signaling pathway, Toll-like receptor (TLR) signaling pathway, containing 34 core targets and 18 corresponding active compounds, were selected for molecular docking. The targets contained in the 4 signaling pathways could be obtained from the Hiplot exported files, and then the signaling pathway-Chinese medicine - disease relationship network was established and visualized and analyzed in Cytoscape_v3.8.2, and the results are shown in Figure 7A–C.

**Figure 8.**
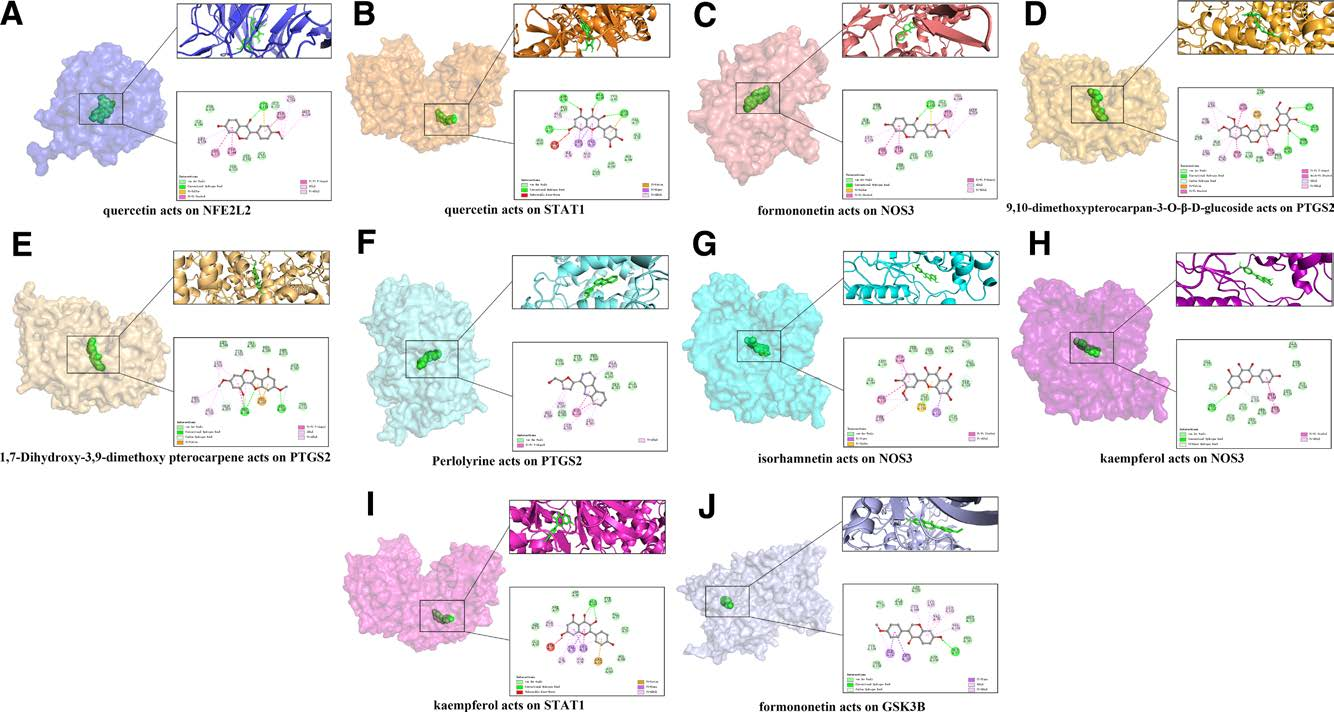
Molecular docking binding of key genes to the active ingredient allows the lowest visualization of 10 groups.

The results of molecular docking are shown in Table 2, after which we selected the 10 combinations with the lowest binding energy for visualization and the results are shown in Figure 8A–J.

**Table 2:** The combination of the best docking model energy.

Compound	Protein	PDB ID	Affinity (kcal/mol)
Quercetin	NFE2L2	4IFL	−9.9
Quercetin	STAT1	6QTJ	−9.9
Formononetin	NOS3	3EAH	−9.8
9,10-dimethoxypterocarpan-3-O-β-D-glucoside	PTGS2	5F19	−9.7
1,7-Dihydroxy-3,9-dimethoxy pterocarpene	PTGS2	5F19	−9.6
Perlolyrine	PTGS2	5F19	−9.6
Isorhamnetin	NOS3	3EAH	−9.6
Kaempferol	NOS3	3EAH	−9.6
Kaempferol	STAT1	6QTJ	−9.6
Formononetin	GSK3B	1Q41	−9.5
Kaempferol	RELA	3QXY	−9.4
Isorhamnetin	PTGS2	5F19	−9.4
Calycosin	GSK3B	1Q41	−9.4
Quercetin	NOS3	3EAH	−9.4
Kaempferol	IKBKB	4KIK	−9.3
Quercetin	RELA	3QXY	−9.2
Quercetin	PTGS2	5F19	−9.2
Isorhamnetin	GSK3B	1Q41	−9.2
Myricanone	GSK3B	1Q41	−9.1
Isorhamnetin	RELA	3QXY	−9
Kaempferol	PTGS2	5F19	−9
Quercetin	MMP3	1HY7	−9
Quercetin	CHUK	5EBZ	−9
Formononetin	PTGS2	5F19	−8.9
Formononetin	JUN	2G01	−8.9
7-O-methylisomucronulatol	NOS3	3EAH	−8.8
Calycosin	PTGS2	5F19	−8.7
3,9-di-O-methylnissolin	NOS3	3EAH	−8.7
Kaempferol	MAPK8	2XRW	−8.6
Kaempferol	JUN	2G01	−8.6
Isorhamnetin	MAPK14	3DT1	−8.6
3,9-di-O-methylnissolin	PTGS2	5F19	−8.5
FA	GSK3B	1Q41	−8.5
Quercetin	MAPK14	3DT1	−8.5
Isorhamnetin	PPARG	6T9C	−8.5
Jaranol	PTGS2	5F19	−8.4
Quercetin	NFKBIA	1IKN	−8.4
Quercetin	JUN	2G01	−8.4
Quercetin	FOS	5PAM	−8.4
Quercetin	PPARG	6T9C	−8.4
(6aR,11aR)-9,10-dimethoxy-6a,11a-dihydro-6H-benzofurano[3,2-c]chromen-3-ol	PTGS2	5F19	−8.3
7-O-methylisomucronulatol	GSK3B	1Q41	−8.3
Quercetin	AKT1	4GV1	−8.3
Quercetin	MMP1	1CGE	−8.2
Kaempferol	PPARG	6T9C	−8.2
Hederagenin	PTGS2	5F19	−8.1
Formononetin	MAPK14	3DT1	−8.1
Quercetin	IFNG	1FYH	−8
Formononetin	PPARG	6T9C	−8
Myricanone	MAPK14	3DT1	−7.9
7-O-methylisomucronulatol	MAPK14	3DT1	−7.8
Calycosin	MAPK14	3DT1	−7.8
Kaempferol	AKT1	4GV1	−7.8
Kaempferol	TNF	2AZ5	−7.7
Quercetin	TNF	2AZ5	−7.7
Myricanone	PPARG	6T9C	−7.7
Calycosin	PPARG	6T9C	−7.6
Bifendate	PTGS2	5F19	−7.5
7-O-methylisomucronulatol	PTGS2	5F19	−7.4
Kaempferol	CASP3	4PS0	−7.4
Quercetin	CASP3	4PS0	−7.4
7-O-methylisomucronulatol	PPARG	6T9C	−7.4
Myricanone	PTGS2	5F19	−7.3
Kaempferol	MMP1	1CGE	−7.3
Quercetin	VCAM1	1VSC	−7.3
Quercetin	MMP9	4JIJ	−7.2
Quercetin	IL1B	5R85	−7.2
Quercetin	CASP9	1JXQ	−7.2
Quercetin	IL6	1N26	−7
Quercetin	SPP1	6J2P	−6.9
Wallichilide	PTGS2	5F19	−6.8
Kaempferol	VCAM1	1VSC	−6.8
Quercetin	TP53	2J21	−6.8
Quercetin	BCL2L1	1MAZ	−6.8
Quercetin	CASP8	4PS1	−6.7
Mandenol	PTGS2	5F19	−6.5
Quercetin	CXCL8	6WZM	−6.2
Quercetin	CCL2	1DOK	−5.9
Quercetin	CXCL10	1O80	−5.9
Formononetin	IL4	2B8U	−5.3
Kaempferol	ICAM1	5MZA	−4.8
Quercetin	ICAM1	5MZA	−4.7

## 4. Discussion

In this study, the network pharmacology research approach helped us to better understand the relationship between the active ingredients in A&L and the potential targets for the treatment of IS. The drug pairs used in TCM to treat diseases often contain multiple herbs and each herb also has multiple chemical small molecules. For studying the relationship between drugs and diseases, network pharmacology can explain their relationship and therapeutic mechanisms well by building various networks, layer by layer, improving the efficiency of drug development and making clinical translation more scientific and rational.^[18]^

A total of 26 active ingredients were identified using the TCMSP database (OB ≥ 30%, DL ≥ 0.18), of which 20 were AM, 7 were LC, and 1 was common to both herbs. Our study of IS was conducted using the DrugBank and GeneCards databases to identify differential genes for this disease. A total of 3848 IS-related differential genes were obtained, followed by a Venn diagram, which yielded 161 common genes. For these genes, we used the STRING database and the MCODE plug-in in Cytoscape 3.8.2 to extract the core network, where the MCODE plug-in automatically categorizes the imported genes and gives them a corresponding score, with higher scores indicating the importance of the core. We selected the first core network with a score of 46.526 and 58 nodes for more in-depth study. The PPI, as well as the GO and KEGG enrichment, is a summary of common genes from the core network to facilitate our understanding of the pathogenesis of disease and the target therapeutic effects of drugs. The results of the GO and KEGG enrichment can be seen in Figure 6, where the GO enrichment shows us important BPs associated with it, such as the response to bacteria-derived molecules, the response to lipopolysaccharides, and the response to biological stimuli.

Four signaling pathways, namely IL-17 signaling pathway, lipid and atherosclerosis, TNF signaling pathway, and TLR signaling pathway, were selected from the KEGG enrichment results. The enrichment results of these 4 signaling pathways were continuously retraced and imported into Cytoscape_v3.8.2 for visualization and analysis, resulting in the identification of 18 small molecules and 34 core targets. These molecules were docked one by one for validation, 82 times in total, and the outputs are shown in Table 2. Normally, a binding energy below −5.0 kcal/mol indicates that the 2 molecules bind well directly, while a binding energy below −7.0 kcal/mol indicates that the 2 molecules have strong binding activity.^[19]^ From Table 2, we can see that most of the docking energies are below −7.0 kcal/mol, which leads us to speculate that A&L can influence the above 4 signaling pathways to treat IS. The 10 combinations with the lowest binding energies are represented in Figure 8 and are shown separately for further analysis.

Among the signaling pathways affected by A&L, IL-17 in the IL-17 signaling pathway is a highly versatile proinflammatory cytokine that is essential for a variety of response processes in the organism.^[20,21]^ Activation of this pathway not only exacerbates the inflammatory response in the brain after ischaemia,^[22]^ but also increases neuronal damage and necrosis through excessive autophagy mediated by the Src-PP2B-mTOR pathway.^[23]^ Controlling the release of inflammatory factors such as IL-17A after cerebral ischemia can effectively protect the brain from secondary damage,^[24]^ reduce apoptosis, and prevent the occurrence of neurodegenerative diseases.^[25]^ TNF is also a major mediator involved in apoptosis and inflammatory responses and is closely associated with sepsis, diabetes, cancer, osteoporosis, rheumatoid arthritis, and inflammatory bowel disease.^[26]^ Experiments have shown that pharmacological agents such as irisin can reduce brain damage and inflammatory injury after IS when they block the TNF signaling pathway.^[27,28]^ The TLR signaling pathway is also currently the focus of research for stroke treatment and plays an important role as a pathway upstream of many other responses.^[29,30]^ For example, the TLR4/nuclear factor-κB signaling pathway activates polarization of microglia, resulting in an excessive inflammatory response.^[31]^ Activation of the IL-17 signaling pathway, TNF signaling pathway, and TLR signaling pathway all lead to adverse immune activation, contributing to the development of central nervous system (CNS) disease and exacerbating the uncontrollable nature of inflammation.^[32]^ KEGG enrichment results include not only the 3 major signaling pathways but also lipid and atherosclerosis as factors. Atherosclerosis as a chronic inflammatory disease has been recognized as one of the main culprits in the rise in stroke-related mortality.^[33,34]^ When lipid-rich plaques form in the walls of the medium and large arteries and increase in size over time, there is a high risk of occlusive thrombosis leading to IS.^[35]^

In summary, A&L has shown great potential in this study for the treatment of stroke, not only with multiple target counterparts and affecting multiple pathways, but also with high binding capacity to receptors and molecular activity even after validation experiments with molecular docking, and AM has shown to have a significant role in stroke prevention by activating blood circulation and resolving blood stasis,^[36]^ as well as the good anti-inflammatory effect of LC.^[37]^ The core network, relevant targets, and signaling pathways obtained in this study could provide a reference for other drugs for the treatment of IS in the future.

However, our study also has certain limitations. For example, the collection of experimental data involves multiple databases, so the accuracy of the information in the databases can affect our conclusions. In this experiment, we have chosen to use computerized molecular docking for initial simulation validation, but network pharmacology also needs basic experimental validation to make its results more accurate and reliable.

## 5. Conclusion

In this study, we found that A&L involves multiple targets and pathways and affects multiple signalling pathways in the treatment of IS. A&L reduces the inflammatory response after IS and avoids the formation of lipid plaques, and the molecular docking results are satisfactory, with the small molecules within it having high activity. A&L demonstrates well the efficacy of TCM in the treatment of IS.

## Author contributions

Conceptualization: Tianyue Wang, Lisheng Chu.

Data curation: Tianyue Wang, Xinyu Jiang, Yanmin Ruan, Lin Li.

Funding acquisition: Lin Li, Lisheng Chu.

Methodology: Tianyue Wang, Xinyu Jiang, Yanmin Ruan.

Project administration: Lisheng Chu.

Software: Xinyu Jiang, Yanmin Ruan.

Supervision: Lin Li, Lisheng Chu.

Visualization: Xinyu Jiang, Yanmin Ruan.

Writing – original draft: Tianyue Wang.

Writing –review and editing: Lin Li, Lisheng Chu.
